# Trends in healthcare visits and antimicrobial prescriptions for acute infectious diarrhea in individuals aged 65 years or younger in Japan from 2013 to 2018 based on administrative claims database: a retrospective observational study

**DOI:** 10.1186/s12879-021-06688-2

**Published:** 2021-09-21

**Authors:** Akane Ono, Kensuke Aoyagi, Yuichi Muraki, Yusuke Asai, Shinya Tsuzuki, Ryuji Koizumi, Toshiaki Azuma, Yoshiki Kusama, Norio Ohmagari

**Affiliations:** 1 AMR Clinical Reference Center, Disease Control and Prevention Center, National Center for Global Health and Medicine, 1-21-1 Toyama, Shinjuku-ku, Tokyo, 162-8655 Japan; 2 Department of Clinical Pharmacoepidemiology, Kyoto Pharmaceutical University, Kyoto, Japan; 3 Disease Control and Prevention Center, National Center for Global Health and Medicine, Tokyo, Japan

**Keywords:** Antimicrobials, Acute infectious diarrhea, Appropriate use, Fosfomycin

## Abstract

**Background:**

The inappropriate use of antimicrobials for acute infectious diarrhea is widespread and leads to the problem of antimicrobial resistance. To improve the use of antimicrobials, it is first necessary to understand the actual situation of diarrheal disease and to identify potential targets for intervention. This study aimed to investigate the recent epidemiological characteristics of and antimicrobial prescriptions for acute infectious diarrhea in Japan.

**Methods:**

This was a retrospective observational study of outpatients aged 0–65 years, separated into children (age 0–17 years) and adults (age 18–65 years), diagnosed with acute infectious diarrhea, using the administrative claims database of the Japan Medical Data Center from 2013 to 2018. We evaluated the number of eligible visits/number of database registrants (defined as the visit rate). The analysis of the antimicrobial prescription rate was restricted to otherwise healthy individuals diagnosed with acute infectious diarrhea alone by excluding patients with multiple disease diagnoses and with medical backgrounds of chronic bowel diseases or immunocompromised conditions. We further classified them by diagnosis of bacterial or nonbacterial acute infectious diarrhea.

**Results:**

The total number of eligible visits for acute infectious diarrhea was 2,600,065. The visit rate, calculated based on the number of eligible visits by database registrants, was higher in children (boys, 0.264; girls, 0.229) than in adults (men, 0.070; women, 0.079), with peaks in early summer and winter. The peaks for visits in adults lagged those of children. In total, 482,484 visits were analyzed to determine the antimicrobial prescription rate; 456,655 (94.6%) were diagnosed with nonbacterial acute infectious diarrhea. Compared with children (boys, 0.305; girls, 0.304), the antimicrobial prescription rate was higher in adults, and there were differences between sexes in adults (men, 0.465; women, 0.408). Fosfomycin and fluoroquinolone were most frequently used for nonbacterial acute infectious diarrhea in children (44.1%) and adults (50.3%), respectively.

**Conclusions:**

These results revealed overprescription of antimicrobials for acute infectious diarrhea in this administrative claims database in Japan and contribute to the development of antimicrobial stewardship strategies and the identification of targets for efficiently reducing inappropriate antimicrobial use.

**Supplementary Information:**

The online version contains supplementary material available at 10.1186/s12879-021-06688-2.

## Background

Antimicrobial resistance (AMR) is a worldwide threat [1] related to the inappropriate use of antimicrobial agents [2, 3], and antimicrobial stewardship strategies are key to tackling the problem [4]. The appropriate use of antimicrobials has been cited as one of the objectives of the Global Action Plan on AMR by the World Health Organization (WHO) and must be addressed globally [5].

The inappropriate use of antimicrobials for acute respiratory tract infection [6, 7] and acute infectious diarrhea, which is common in large numbers of patients [8, 9] is an inevitable problem. These diseases are mostly caused by viruses and empirical antimicrobials are not necessary for otherwise healthy patients (here, ‘otherwise healthy’ indicates that except for acute infectious diarrhea, the patients had no other medical conditions requiring treatment), particularly in developed countries [10, 11]. A previous Japanese study analyzing data from 2012 to 2015 showed that 40.5% of antimicrobials were prescribed for viral upper respiratory infections, 25.8% for gastrointestinal infections, and 6.7% for nonbacterial gastrointestinal infections [12]. In response to the Global Action Plan, similar to other nations, the Government of Japan prepared the National Action Plan on AMR in 2016 [13] and the Ministry of Health, Labour, and Welfare (MHLW) of Japan drafted the Manual of Antimicrobial Stewardship in 2017, particularly regarding acute respiratory tract infection and acute infectious diarrhea [14].

Although several studies have examined the role of antimicrobial stewardship in acute respiratory tract infections [6, 15, 16], few studies have examined this issue in relation to diarrhea in Japan, excluding a database study in children [17] and in all ages [12], but both of these studies were based on data up to 2015 and did not compare data between before and after the preparation of the National Action Plan and the manual. To implement measures for national antimicrobial stewardship for acute infectious diarrhea, understanding is required of the epidemiological characteristics of and antimicrobial prescriptions for acute infectious diarrhea. Identification of areas of inappropriate antimicrobial use would help to promote efficient antimicrobial stewardship. Therefore, we conducted a retrospective observational study using an administrative claims database, including data obtained before and after the preparation of the National Action Plan and the manual.

## Methods

### Study design and data source

We retrospectively analyzed the administrative claims database of patients diagnosed with acute infectious diarrhea between January 1, 2013, and December 31, 2018, using a database from the Japan Medical Data Center (JMDC) Co., Ltd. (Tokyo, Japan). The JMDC Claims Database is a nationwide electronic database of completely anonymized individual records extracted from insurance societies throughout Japan. Its population comprises employees and their family members; as of June 2018, its cumulative population size is about 5.8 million [18]. Because of the nature of the database population, persons of working age predominate and few data are available on persons older than 65 years. This database contains patients’ sex, year and month of birth, date of diagnosis, diagnosis codes, prescriptions, medical procedures, laboratory tests (results are not included) and other medical services, and inpatient or outpatient status. Diagnosis codes are recorded as International Classification of Diseases, 10th Revision (ICD-10) codes. Prescribed medications are recorded using the codes of the standard Anatomic Therapeutic Chemical Classification System (ATC codes). In addition, the route of drug administration is coded separately as oral, injectable, topical, dental, and others. In this study, we extracted only the code for oral administration.

### Study population and definition of acute infectious diarrhea

Eligible patients were selected according to the ICD-10 code-identified diagnosis. Acute infectious diarrhea was defined in this study as any diarrhea resulting from any kind of microorganism and food. We chose the ICD-10 codes corresponding to the diagnosis of intestinal infection based on Clinical Classification Software Refined because these codes are regarded to be more clinically meaningful [19]. The ICD-10 codes selected for acute infectious diarrhea were categorized into A00, A01, A02.0, A02.9, A03, A04, A05, A18.3, A21.3, A22.2, and T62.9 as clinically suspected bacterial acute infectious diarrhea and A06.0, A06.2, A06.9, A07, A08, A09, and B37.8 as nonbacterial acute infectious diarrhea, meaning that the use of antimicrobials would not be appropriate (see Additional file 1: Table S1). If a single visit was associated with several of the above ICD-10 codes for acute infectious diarrhea diagnosis and had both bacterial and nonbacterial codes, the patient was regarded as having bacterial infection and was categorized in the appropriate use group.

### Inclusion and exclusion criteria

**Fig. 1:**
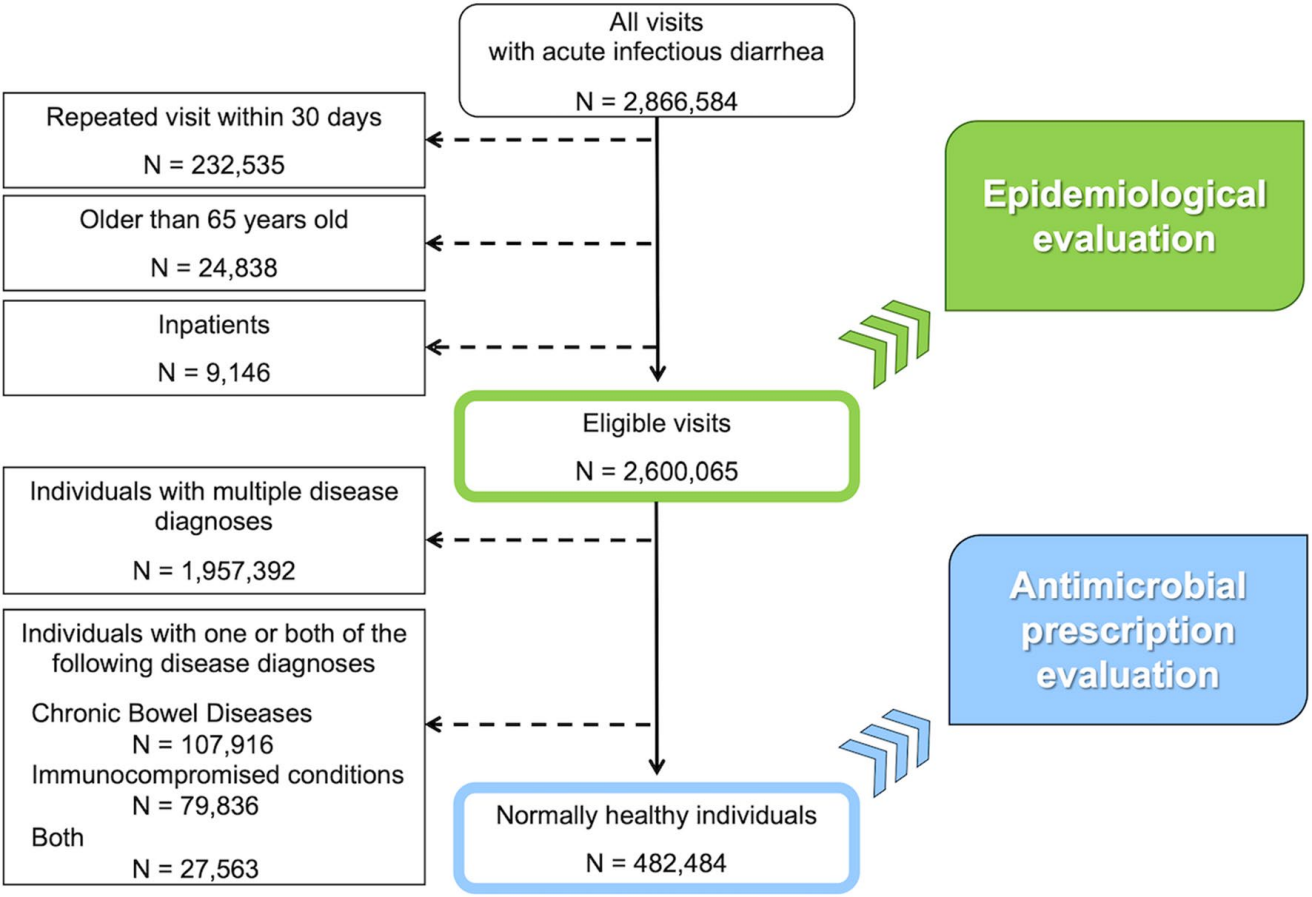
Procedures for the inclusion and exclusion of acute infectious diarrhea, 2013–2018. N: number of eligible visits

Patients’ visits due to acute infectious diarrhea were identified by the above ICD-10 codes (Fig. 1). To avoid repeated visits for a single episode of acute infectious diarrhea, we determined if the same codes had been used in the previous 30 days; if so, we included only the first one. We used a time limit of 30 days to exclude repeat visits for follow-up consultations [18]. When examining the prescription of oral antimicrobials for acute infectious diarrhea, we limited the visits with diagnosis to only those for acute infectious diarrhea alone as defined in this study in order to exclude the prescription of antimicrobials for other diseases.

We excluded patients older than 65 years old because the JMDC database has few data on this population, as well as patients who were hospitalized because our aim was to identify visits in primary care. The study population was categorized into groups aged 0–17 years old (children) and 18–65 years old (adults). The age for the former group was defined based on the Convention on the Rights of the Child by the United Nations [20] and the pediatric terminology proposed by the Eunice Kennedy Shriver National Institute of Child Health and Human Development [6, 21].

To evaluate the use of antimicrobials in otherwise healthy patients for who an antimicrobial prescription was not justified at the first visit, we stratified patients by medical background—otherwise healthy, chronic bowel disease, and immunocompromised conditions—and excluded the latter two backgrounds in addition to those patients with multiple disease diagnoses. The definitions of these three backgrounds are shown in Additional file 1: Table S2.

### Epidemiological evaluation of outpatients aged 0–65 years with acute infectious diarrhea

We extracted the number of eligible visits for acute infectious diarrhea by year and month, by age group, and by sex within the 2013 to 2018 period. The rate of the number of eligible visits/number of JMDC registrants as of October of each year from 2013 to 2018 was defined as the visit rate and used in the analysis.

To assess the seasonality of acute infectious diarrhea, we plotted the visit rate as a monthly trend. In this study, we defined March–May as spring, June–August as summer, September–November as autumn, and December–February as winter.

### Evaluation of antimicrobial prescriptions to otherwise healthy outpatients aged 0–65 years with acute infectious diarrhea only

In only otherwise healthy individuals, bacterial and nonbacterial acute infectious diarrhea were evaluated separately with respect to antimicrobial prescription rates. The antimicrobial prescription rate was calculated and evaluated as the number of visits with antimicrobial prescriptions divided by the number of visits targeted for the evaluation of antimicrobial prescriptions. The categories of selected antimicrobials and monthly trends in antimicrobial prescription rates were analyzed specifically for nonbacterial acute infectious diarrhea.

Information on antimicrobials was extracted by ATC codes J01, A07AA, and P01AB, based on the definition of antimicrobials by the WHO surveillance program [22]. Data were collected at ATC level 5 and were arbitrarily classified into seven categories as penicillins (J01CA, J01CE, and J01CR), cephalosporins (J01DB, J01DC, and J01DD), macrolides (J01FA), fluoroquinolones (J01MA), fosfomycin (J01XX), metronidazole (J01XD and P01AB; in the relevant data, J01XX was only fosfomycin and J01XD and P01AB were only metronidazole), and others (A07AA, J01AA, J01BA, J01DH, J01DI, J01ED, J01EE, J01FF, and J01MB), based on ATC levels 4 and 5 (see Additional file 1: Table S3).

### Statistical analysis

We evaluated the median and interquartile range for continuous variables. We also calculated numbers and percentages for the categorical variables.

The visit rate and antimicrobial prescription rate as defined in this study were calculated. The visit rate was calculated by dividing the number of eligible visits by the total number of JMDC registrants as of October of each year from 2013 to 2018. The number of eligible visits was defined as the “epidemiological evaluation” (Fig. 1). The antimicrobial prescription rate was calculated using the results of the “antimicrobial prescription evaluation” (Fig. 1) by dividing the number of visits with antimicrobial prescriptions by the number of visits targeted for the evaluation of antimicrobial prescriptions.

## Results

### Trends in acute infectious diarrhea in the population aged 0–65 years

**Fig. 2:**
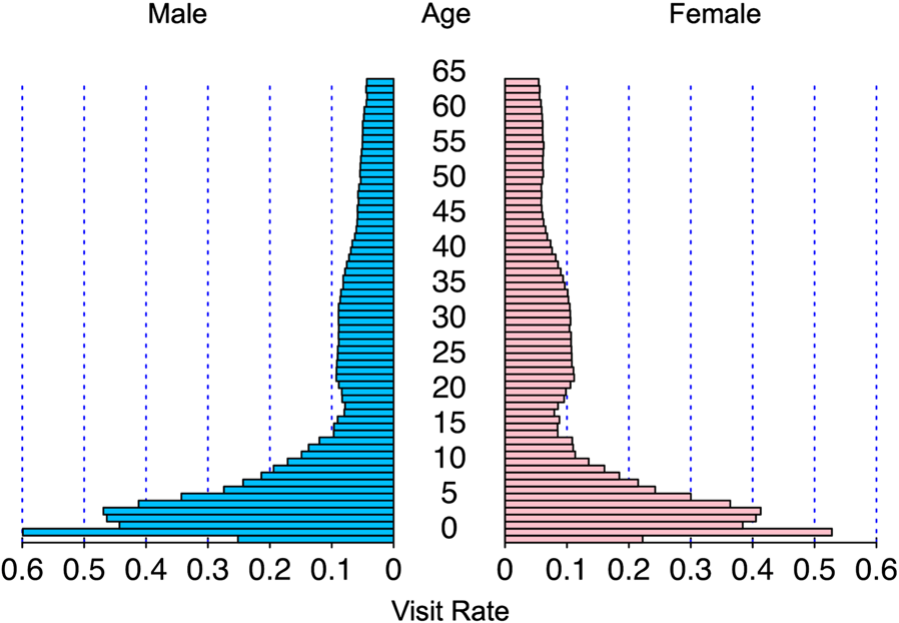
Age distribution of the visit rate by sex in patients aged 0–65 years, 2013–2018. The visit rate was calculated as follows: Visit rate = number of eligible visits/number of JMDC registrants as of October of each year from 2013 to 2018

The total number of eligible visits with acute infectious diarrhea for this study was 2,600,065 (male:female = 54.5%:45.5%). The number of children was 1,367,176 (52.6%), and the number of adults was 1,232,889 (47.4%) (see Additional file 1: Table S4). Median age was 15 (interquartile range, 5–35) years. The age distribution of the visit rate (shown in Fig. 2) was bimodal. Most visits were concentrated in patients 1 to 5 years old, and there was a slight peak in patients in their 20 s and 30 s. The former peak was slightly higher for boys, whereas the latter peak was slightly higher for women. During the 6-year study period, the age distribution was generally similar. The visit rate in each year by sex and age group is shown in Table 1. The visit rate was higher among children than among adults. Among children, the visit rate was higher in boys (0.264) than in girls (0.229) but, among adults, the rate was higher in women (0.079) than in men (0.070). It remained almost unchanged for 6 years in both age groups and sexes.

**Table 1:** Visit rates for acute infectious diarrhea in each year by age group and sex

Age group (years)	Sex	2013	2014	2015	2016	2017	2018	Total
0–17	Male	0.303	0.300	0.273	0.280	0.234	0.227	0.264
	Female	0.263	0.258	0.237	0.244	0.204	0.199	0.229
18–65	Male	0.076	0.076	0.071	0.074	0.063	0.066	0.070
	Female	0.088	0.087	0.080	0.084	0.071	0.073	0.079

The visit rate was calculated as follows: Visit rate = number of eligible visits/number of JMDC registrants as of October of each year from 2013 to 2018

**Fig. 3:**
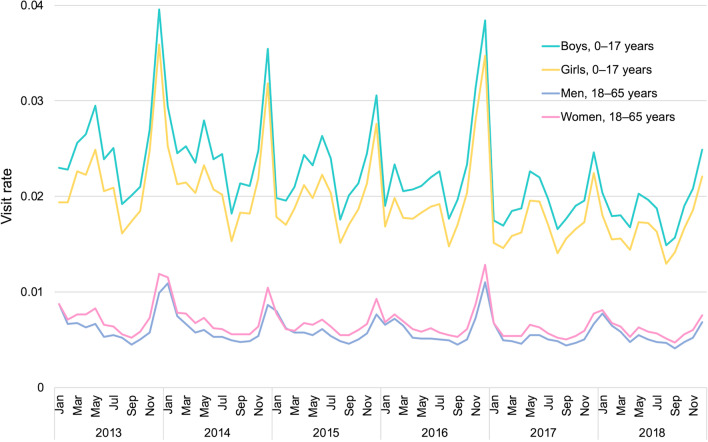
Monthly change in the visit rate for acute infectious diarrhea by age group and sex. The visit rate was calculated as follows: Visit rate = number of eligible visits/number of JMDC registrants as of October of each year from 2013 to 2018

The total number of acute infectious diarrheal visits per month showed seasonal variations, with peaks between November and January and between May and July each year during the study period; there was a particularly large peak in winter and the largest increase was in 2016 (Fig. 3). The seasonal change was similar for both age groups. There were generally fewer visits in adults than in children, with the peak in adults lagging behind children by nearly 1 month. The visit rates were higher for boys than for girls and for women than for men over the 6-year period.

In this study population, the total number of diagnoses was 2,667,448, which was 1.03 times the total number of visits, 2,600,065, meaning that almost all visits had a single diagnosis. The vast majority (93.6%) were classified as infectious gastroenteritis and colitis, unspecified (A09), and only 4.4% were classed as bacterial infectious diarrhea as defined in this study (see Additional file 1: Table S5).

### Antimicrobial prescriptions for nonbacterial acute infectious diarrhea in otherwise healthy individuals

Of the 2,600,065 visits, we excluded those with multiple disease diagnoses, chronic bowel diseases, or immunocompromised conditions (see Additional file 1: Table S2). We then analyzed otherwise healthy patients diagnosed with acute infectious diarrhea alone (482,484; 18.6%) (Fig. 1); 276,766 were children (57.4%; boys:girls = 56.4%:43.6%), whereas 205,718 were adults (42.6%; men:women = 60.2%:39.8%) (see Additional file 1: Table S6).

Overall, 94.6% of otherwise healthy patients with acute infectious diarrhea alone were diagnosed with nonbacterial acute infectious diarrhea. Even though none of these patients had bacterial acute infectious diarrhea, the antimicrobial prescription rate for nonbacterial acute infectious diarrhea was 0.305 for boys and 0.304 for girls, but 0.465 for men and 0.408 for women (Table 2).

**Table 2:** Number of visits and antimicrobial prescriptions for acute infectious diarrhea among otherwise healthy patients, 2013–2018

	Age group	Sex	Visits	AMP	Rate
Bacterial	0–17 years	Male	7291	5853	0.803
		Female	5177	4170	0.805
	18–65 years	Male	8814	7014	0.796
		Female	4547	3538	0.778
	Total		25,829	20,575	0.797
Nonbacterial	0–17 years	Male	148,732	45,309	0.305
		Female	115,566	35,087	0.304
	18–65 years	Male	115,093	53,482	0.465
		Female	77,264	31,546	0.408
	Total		456,655	165,424	0.362

The antimicrobial prescription rate was calculated as follows: Rate = number of visits with an antimicrobial prescription/number of visits targeted for the evaluation of antimicrobial prescriptions

Visits: number of visits; AMP: number of eligible visits with an antimicrobial prescription; Rates: antimicrobial prescription rate

**Fig. 4:**
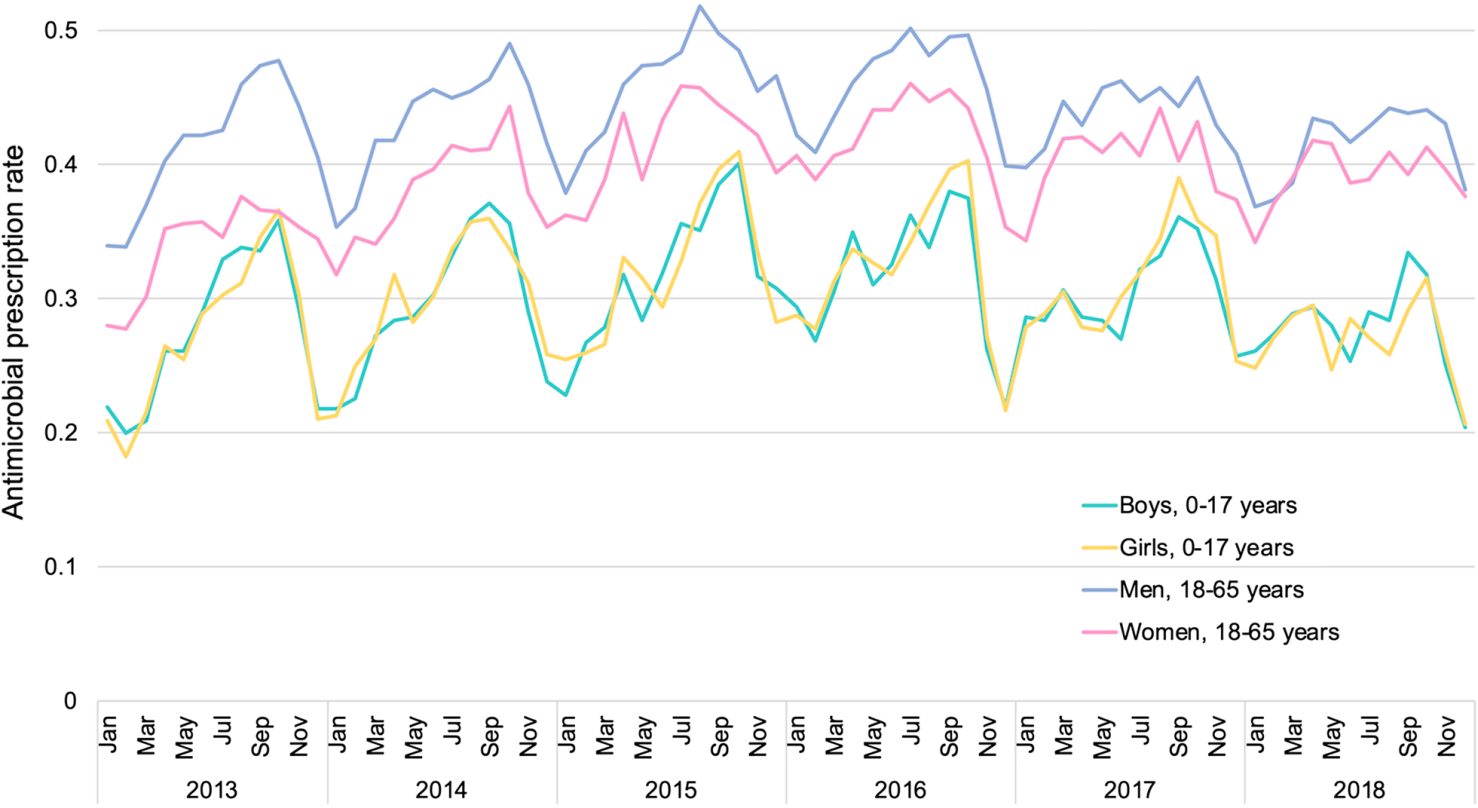
Monthly antimicrobial prescription rates among otherwise healthy patients with nonbacterial acute infectious diarrhea

Among antimicrobial prescriptions for nonbacterial acute infectious diarrhea, the antimicrobial prescription rate showed an upward trend until 2015 and then a downward trend. The monthly antimicrobial prescription rate trended higher in summer and lower in winter. In all years of the study period, the antimicrobial prescription rates were higher in adults than in children. There was little difference between boys and girls, but there was a difference between men and women (Fig. 4).

**Fig. 5:**
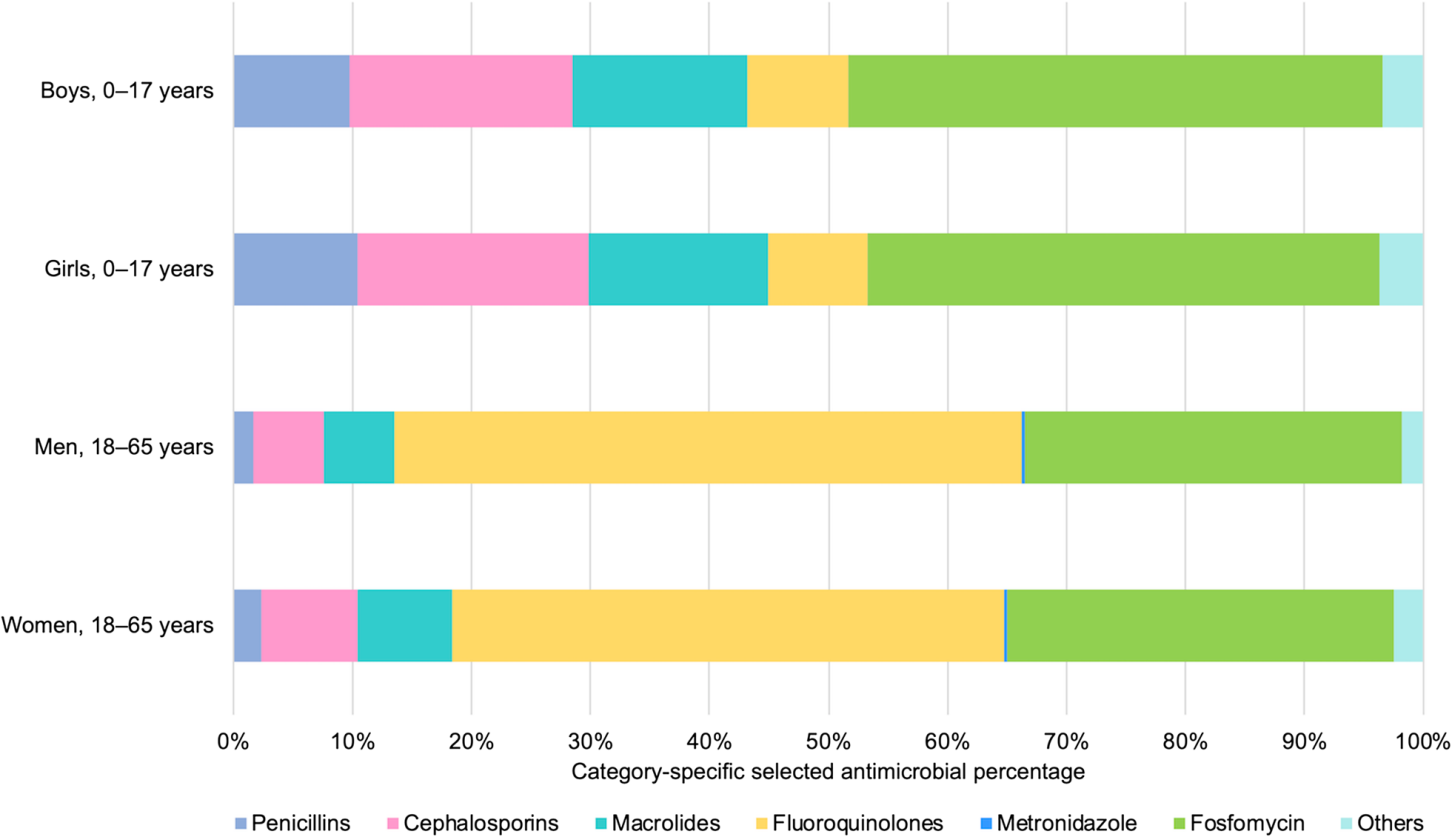
Proportion of category-specific selected antimicrobials among otherwise healthy patients with nonbacterial acute infectious diarrhea, 2013–2018. Details on the antimicrobial category are shown in Additional file 1: Table S3

The most frequent antimicrobial prescription in the category of our study was fosfomycin (37.7%), followed by fluoroquinolones (30.5%). Fosfomycin was the most frequent antimicrobial prescription overall among children (44.1%) and was prescribed to 44.9% of boys and 43.1% of girls. Among adults, fluoroquinolones were the most frequently prescribed antimicrobials (50.3%), followed by fosfomycin (32.0%). The percentage of fluoroquinolones was 52.6% for men and 46.4% for women, whereas that of fosfomycin was 31.8% for men and 32.5% for women (Fig. 5).

## Discussion

In this study, we determined the epidemiological characteristics of and antimicrobial prescriptions for acute infectious diarrhea. To our knowledge, no studies have evaluated antimicrobial prescriptions for acute infectious diarrhea in the Japanese population, except for one database study in children [17]. Therefore, we believe this study is the first to show the Japanese trend of acute infectious diarrhea based on the JMDC database.

As reported in other countries, the visit rate was highest among children younger than 5 years of age [23, 24]. Bimodal seasonal trends were observed with peaks around June and December. There was an outbreak of norovirus in winter 2016, the second largest outbreak in the past 10 seasons [25], which produced a large number of acute infectious diarrhea patients. The peak antimicrobial prescription rate was observed in the summer season, and we believe that this can be explained by the increase in obviously viral infectious diarrhea such as noroviral disease in the winter season. A similar pattern was found in a previous study of upper respiratory infections in Japan [18]. The peaks in adults lagged those in children by about 1 month. From this, we speculate that acute infectious diarrhea spreads from children to parents, and our finding that the population aged 20 s to 40 s showed a slight peak in the age distribution supports our speculation.

Visit rates were higher in boys than in girls and in women than in men. A higher visit rate was identified in women in a patient survey by the MHLW, with data obtained from the Statistics Bureau of Japan [26] and in studies from other countries [23, 24, 27, 28]. The reason is considered to be that women generally have more contact with their children than men, increasing child-to-mother transmission. In the age distribution, the peak in the 20 s to 40 s population showed an appreciable sex difference. Meanwhile, we could not find any plausible reason for the sex difference in children, although the same tendency was observed in a previous study [29]. However, we consider that another approach, for example; a questionnaire survey, can explore the reasons of the difference.

Antimicrobial prescription rates increased from 0.342 in 2013 to 0.407 in 2016, but decreased thereafter (0.406 in 2017 and 0.386 in 2018). This trend could be attributable to the development of the National Action Plan on AMR [13] and various subsequent countermeasures [30]. The National Action Plan does not restrict antimicrobial use but aims to improve physicians’ awareness of antimicrobial use. In addition, the Manual of Antimicrobial Stewardship [14] explains how antimicrobials should be used for diarrheal diseases, which would promote judicious antimicrobial use. However, antimicrobial prescriptions for nonbacterial acute infectious diarrhea remained high even in 2018, exceeding 0.35 in adults. The antimicrobial prescription rate was higher in adults than in children. We observed the same pattern in a previous study of nonbacterial acute respiratory tract infections; therefore, educational approaches to physicians in internal medicine are required [18]. The prescription rate was higher in men than in women. The reason is unclear, but we hypothesize that there may be a difference in health literacy for antimicrobials between men and women. In this regard, we are currently conducting another survey.

Fluoroquinolones, which are recommended for the treatment of community-acquired bacterial enteritis [11, 31], were the most prescribed antimicrobials in adults. Meanwhile, the high proportion of fosfomycin prescriptions, irrespective of whether to children or adults, seems to be a unique characteristic to Japan. We consider that this might be because Japanese physicians are accustomed to prescribing fosfomycin, and this tendency might have been strengthened by two studies conducted in Japan, which reported that fosfomycin decreased the incidence of hemolytic-uremic syndrome in enterohemorrhagic *Escherichia coli* enteritis [32, 33]. However, this does not justify indiscriminate prescriptions for acute infectious diarrhea because nonbacterial acute infectious diarrhea is far more frequent than bacterial acute infectious diarrhea. Appropriate diagnosis and the prescribing of antimicrobials only when they are needed are important for promoting antimicrobial stewardship regarding acute infectious diarrhea. The Manual of Antimicrobial Stewardship states that acute diarrhea, whether bacterial or nonbacterial in origin, often resolves spontaneously, and that symptomatic treatment is more important than antimicrobial prescription [14].

This study has several limitations. First, because of the retrospective and administrative claims database nature of the study, clinical information is not available. Therefore, it is impossible to know the rationale for the antimicrobial prescriptions and the adherence of patients who received antimicrobials. Further research is needed to evaluate these issues. Second, because of the characteristics of the database, specifically the unevenly selected population, there are biases in the social background and age distribution of the population in this study. Therefore, the results of this study cannot be applied to patients older than 65 years and therefore cannot generalize to the entire population in Japan. However, many previous studies have used the data as being representative of Japan [34]. We believe that we can provide useful information on the epidemiology of acute diarrhea and inappropriate antimicrobial prescriptions, as these studies did. Additionally, despite the social background bias, the 2017 MHLW survey showed a similar estimation of acute infectious diarrhea visits to our results in a population younger than 65 years, so our results can be generalized to that population. Third, due to a lack of the disease name or code corresponding to traveler’s diarrhea, we cannot evaluate the use of antimicrobials for these patients. However, because the numbers of such patients are small, this limitation is expected to have very little effect on the results of this study. Fourth, precise diagnosis cannot be guaranteed and a diagnosis of bacterial diarrhea does not necessarily mean that the patient has bacterial diarrhea. Yet, despite these limitations, this claims database study identified the targets of antimicrobial stewardship for acute infectious diarrhea by separately assessing sex and age groups. This study enables the more efficient implementation of AMR measures.

## Conclusions

This study revealed that acute infectious diarrhea occurred more commonly in children than in adults. Seasonal peaks of the incidence in children were observed 1 month prior to those in adults. Acute infectious diarrhea occurred more commonly in boys than in girls but less commonly in men than in women. We also found that the rate of antimicrobial prescriptions to nonbacterial acute infectious diarrhea exceeded 0.35 even in 2018 and was higher in adult patients than in pediatric patients. Among adults, the rate was higher in male patients than in female patients. The most frequently prescribed antimicrobial categories were fluoroquinolone in adults and fosfomycin in children. Judicious use of antimicrobials for acute infectious diarrhea is necessary to combat AMR and support antimicrobial stewardship strategies. These results of this study revealed widespread inappropriate antimicrobial prescribing for acute infectious diarrhea in Japan, and contribute to the development of antimicrobial stewardship strategies and the identification of targets for efficiently reducing inappropriate antimicrobial use. Claims databases are suitable for identifying these targets and further research should be conducted in various areas of medicine and various countries to reveal malpractices in antimicrobial prescriptions and to develop effective countermeasures to ensure appropriate use of antimicrobials.

## Supplementary Information

**Additional file 1: Table S1.** Definitions of the classification for bacterial or nonbacterial acute infectious diarrhea based on ICD-10 code. **Table S2.** Definitions of medical backgrounds excluded in the evaluation of antimicrobial use. **Table S3.** Definitions of antimicrobial categories based on ATC codes and the category name (capitalized) in this study. **Table S4.** Total numbers of annual visits with acute infectious diarrhea by age group and sex. **Table S5-1.** Numbers and proportions of acute infectious diarrhea to total visits for each diagnosis code by age group and in total, N (%).**Table S5-2.** Numbers and visit rate of acute infectious diarrhea based on diagnosis code by age group and in total, N (visit rates). **Table S6.** Numbers of annual visits among normally healthy patients with acute infectious diarrhea alone by age group and sex.

## Data Availability

The data that support the findings of this study are available from JMDC and the datasets used and analyzed in this study are available from the corresponding author on reasonable request.

## Abbreviations

AMR: Antimicrobial resistance
WHO: World Health Organization
MHLW: Ministry of Health, Labour and Welfare
JMDC: Japan Medical Data Center
ICD-10: International Classification of Diseases, 10th Revision
ATC codes: Codes of the standard Anatomic Therapeutic Chemical Classification System
