# Supplementary material for: Trends in healthcare visits and antimicrobial prescriptions for acute infectious diarrhea in individuals aged 65 years or younger in Japan from 2013 to 2018 based on administrative claims database: a retrospective observational study
Source: BMC Infect Dis. 2021 Sep 21;21:983. doi: 10.1186/s12879-021-06688-2 (PMC8454076; doi:10.1186/s12879-021-06688-2)
Supplement: Supplementary file 1 — Additional file 1: Table S1. Definitions of the classification for bacterial or nonbacterial acute infectious diarrhea based on ICD-10 code. Table S2. Definitions of medical backgrounds excluded in the evaluation of antimicrobial use. Table S3. Definitions of antimicrobial categories based on ATC codes and the category name (capitalized) in this study. Table S4. Total numbers of annual visits with acute infectious diarrhea by age group and sex. Table S5-1. Numbers and proportions of acute infectious diarrhea to total visits for each diagnosis code by age group and in total, N (%).Table S5-2. Numbers and visit rate of acute infectious diarrhea based on diagnosis code by age group and in total, N (visit rates). Table S6. Numbers of annual visits among normally healthy patients with acute infectious diarrhea alone by age group and sex. [file 12879_2021_6688_MOESM1_ESM.docx]

***Additional Tables; S1–S6***

**Table S1: Definitions of the classification for bacterial or nonbacterial acute infectious diarrhea based on ICD-10 code**

|  | **ICD-10 code** | **ICD-10 code description** |
| --- | --- | --- |
| Bacterial | A00 | Cholera |
|  | A01 | Typhoid and paratyphoid fevers |
|  | A02.0 | Salmonella enteritis |
|  | A02.9 | Salmonella infection, unspecified |
|  | A03 | Shigellosis |
|  | A04 | Other bacterial intestinal infections |
|  | A05 | Other bacterial foodborne intoxications, not elsewhere classified |
|  | A18.3 | Tuberculosis of intestines, peritoneum, and mesenteric glands |
|  | A21.3 | Gastrointestinal tularemia |
|  | A22.2 | Gastrointestinal anthrax |
|  | T62.9 | Toxic effect of unspecified noxious substance eaten as food |
| Nonbacterial | A06.0 | Acute amebic dysentery |
|  | A06.2 | Amebic nondysenteric colitis |
|  | A06.9 | Amebiasis, unspecified |
|  | A07 | Other protozoal intestinal diseases |
|  | A08 | Viral and other specified intestinal infections |
|  | A09 | Infectious gastroenteritis and colitis, unspecified |
|  | B37.8 | Candidiasis of other sites |

ICD-10, International Classification of Diseases, 10th Revision.

**Table S2: Definitions of medical backgrounds excluded in the evaluation of antimicrobial use**

| **Code type** | **Code** | **Code description** |
| --- | --- | --- |
| **Chronic bowel disease** | | |
| ICD-10 | K50 | Crohn's disease [regional enteritis] |
| ICD-10 | K51 | Ulcerative colitis |
| ICD-10 | K52 | Other and unspecified noninfective gastroenteritis and colitis |
| ICD-10 | K58 | Irritable bowel syndrome |
| ICD-10 | K59 | Other functional intestinal disorders |
| ICD-10 | K90 | Intestinal malabsorption |
| ICD-10 | K91 | Intraoperative and postprocedural complications and disorders of digestive system, not elsewhere classified |
| **Immunocompromised condition** | | |
| Procedure | K921 | Hematopoietic stem cell transplant |
| Procedure | K697-5, K697-7, K780, K780-2, K709-3, K709-5, K514-6, K514-4, K605-2, K605-4 | Solid organ transplantation |
| ICD-10 | C00-80 | Solid organ malignancies |
| ICD-10 | C81-96 | Hematological malignancies |
| ICD-10 | M059, M060, M068 | Rheumatoid arthritis |
| ICD-10 | M32 | Systemic lupus erythematosus |
| ICD-10 | K50, 51 | Inflammatory bowel disease |
| ICD-10 | L40, M070-073, M090 | Psoriasis |
| ICD-10 | G35 | Multiple sclerosis |
| ICD-10 | M353, M315 | Polymyalgia rheumatica |
| ICD-10 | E063 | Autoimmune thyroiditis |
| ICD-10 | M30, M31 | Vasculitis |
| ICD-10 | I01, J990, J991, L10, L11, L12, L405, L920, M061, M33, M34, M350, M351, M352, M359, M36, M941 | Other autoimmune/collagen-connective tissue diseases  Chronic inflammatory diseases (autoimmune diseases) |
| ICD-10 | B20-B24 | Human immunodeficiency virus |
| ICD-10 | N18 | Chronic kidney disease and end-stage renal disease |
| Procedure | J038, J038-2, J042, K780, K780-2 |  |
| ICD-10 | D60, D61, D70, D71, D72, D76.1, D80, D81, D82, D83, D84, D89, G113 | Congenital immune deficiency |
| ICD-10 | E10 | Type 1 diabetes mellitus |

Otherwise healthy patients were extracted as patients without any chronic bowel diseases or immunocompromised conditions.

Chronic bowel diseases were identified by ICD-10 codes K50, K51, K52, K58, K59, K90, and K91, diagnosed in this study period.

The definition of immunocompromised conditions is based on prior studies^1, 2^ and 17 conditions were identified by the means of ICD-10 codes and/or procedure codes.

ICD-10, International Classification of Diseases, 10th Revision.

**Table S3: Definitions of antimicrobial categories based on ATC codes and the category name (capitalized) in this study**

| **Antimicrobial category** | **ATC codes included** |
| --- | --- |
| Penicillins | J01CA, J01CE, J01CR |
| Cephalosporins | J01DB, J01DC, J01DD |
| Macrolides | J01FA |
| Fluoroquinolones | J01MA |
| Fosfomycin | J01XX |
| Metronidazole | J01XD, P01AB |
| Others | A07AA, J01AA, J01BA, J01DH, J01DI, J01ED, J01EE, J01FF, J01MB |

ACT, Anatomic Therapeutic Chemical Classification System.

**Table S4: Total numbers of annual visits with acute infectious diarrhea by age group and sex**

| **Age group** | **Sex** | **2013** | **2014** | **2015** | **2016** | **2017** | **2018** | **Total** |
| --- | --- | --- | --- | --- | --- | --- | --- | --- |
| 0–17 years | Male | 102,235 | 106,477 | 125,696 | 145,585 | 137,541 | 132,365 | 749,899 |
|  | Female | 83,607 | 86,522 | 103,427 | 119,931 | 113,781 | 110,009 | 617,277 |
| 18–65 years | Male | 82,770 | 86,890 | 108,964 | 129,846 | 126,607 | 132,137 | 667,214 |
|  | Female | 70,132 | 72,383 | 92,642 | 111,677 | 108,690 | 110,151 | 565,675 |

**Table S5-1: Numbers and proportions of acute infectious diarrhea to total visits for each diagnosis code by age group and in total, N (%)**

| **ICD-10 code** | **Code description** | **0–17 years** | | **18–65 years** | | **0–65 years** | |
| --- | --- | --- | --- | --- | --- | --- | --- |
|  | **Bacterial infections** | **43,013** | (3.07) | **75,093** | (5.92) | **118,106** | (4.43) |
| A00 | Cholera | 6 | (0.00) | 18 | (0.00) | 24 | (0.00) |
| A01 | Typhoid and paratyphoid fevers | 11 | (0.00) | 93 | (0.01) | 104 | (0.00) |
| A02 | Salmonella enteritis/Salmonella infection, unspecified | 807 | (0.06) | 1,316 | (0.10) | 2,123 | (0.08) |
| A03 | Shigellosis | 14 | (0.00) | 146 | (0.01) | 160 | (0.01) |
| A04 | Other bacterial intestinal infections | 40,748 | (2.91) | 68,311 | (5.39) | 109,059 | (4.09) |
| A05 | Other bacterial foodborne intoxications, not elsewhere classified | 382 | (0.03) | 663 | (0.05) | 1,045 | (0.04) |
| A18 | Tuberculosis of intestines, peritoneum, and mesenteric glands | 46 | (0.00) | 1,092 | (0.09) | 1,138 | (0.04) |
| A21 | Gastrointestinal tularemia | 0 | (0.00) | 0 | (0.00) | 0 | (0.00) |
| A22 | Gastrointestinal anthrax | 0 | (0.00) | 0 | (0.00) | 0 | (0.00) |
| T62 | Toxic effect of unspecified noxious substance eaten as food | 999 | (0.07) | 3,454 | (0.27) | 4,453 | (0.17) |
|  | **Nonbacterial infections** | **1,357,031** | (96.93) | **1,192,311** | (94.08) | **2,549,342** | (95.6) |
| A06 | Acute amebic dysentery/Amebic nondysenteric colitis/Amebiasis, unspecified | 36 | (0.00) | 1,374 | (0.11) | 1,410 | (0.05) |
| A07 | Other protozoal intestinal diseases | 5 | (0.00) | 81 | (0.01) | 86 | (0.00) |
| A08 | Viral and other specified intestinal infections | 37,073 | (2.65) | 10,973 | (0.87) | 48,046 | (1.80) |
| A09 | Infectious gastroenteritis and colitis, unspecified | 1,319,362 | (94.24) | 1,176,558 | (92.83) | 2,495,920 | (93.57) |
| B37 | Candidiasis of other sites | 555 | (0.04) | 3,325 | (0.26) | 3,880 | (0.15) |
|  | **Total** | **1,400,044** | (100) | **1,267,404** | (100) | **2,667,448** | (100) |

ICD-10, International Classification of Diseases, 10th Revision.

**Table S5-2: Numbers and visit rate of acute infectious diarrhea based on diagnosis code by age group and in total, N (visit rates)**

| **ICD-10 code** | **Code description** | **0–17 years** | | **18–65 years** | | **0–65 years** | |
| --- | --- | --- | --- | --- | --- | --- | --- |
|  | **Bacterial infections** | **43,013** | 0.008 | **75,093** | 0.004 | **118,106** | 0.005 |
| A00 | Cholera | 6 | < 0.001 | 18 | < 0.001 | 24 | < 0.001 |
| A01 | Typhoid and paratyphoid fevers | 11 | < 0.001 | 93 | < 0.001 | 104 | < 0.001 |
| A02 | Salmonella enteritis/Salmonella infection, unspecified | 807 | < 0.001 | 1,316 | < 0.001 | 2,123 | < 0.001 |
| A03 | Shigellosis | 14 | < 0.001 | 146 | < 0.001 | 160 | < 0.001 |
| A04 | Other bacterial intestinal infections | 40,748 | 0.007 | 68,311 | 0.004 | 109,059 | 0.005 |
| A05 | Other bacterial foodborne intoxications, not elsewhere classified | 382 | < 0.001 | 663 | < 0.001 | 1,045 | < 0.001 |
| A18 | Tuberculosis of intestines, peritoneum, and mesenteric glands | 46 | < 0.001 | 1,092 | < 0.001 | 1,138 | < 0.001 |
| A21 | Gastrointestinal tularemia | 0 | 0 | 0 | 0 | 0 | 0 |
| A22 | Gastrointestinal anthrax | 0 | 0 | 0 | 0 | 0 | 0 |
| T62 | Toxic effect of unspecified noxious substance eaten as food | 999 | < 0.001 | 3,454 | < 0.001 | 4,453 | < 0.001 |
|  | **Nonbacterial infections** | **1,357,031** | 0.245 | **1,192,311** | 0.071 | **2,549,342** | 0.115 |
| A06 | Acute amebic dysentery/Amebic nondysenteric colitis/Amebiasis, unspecified | 36 | < 0.001 | 1,374 | < 0.001 | 1,410 | < 0.001 |
| A07 | Other protozoal intestinal diseases | 5 | < 0.001 | 81 | < 0.001 | 86 | < 0.001 |
| A08 | Viral and other specified intestinal infections | 37,073 | 0.007 | 10,973 | < 0.001 | 48,046 | 0.002 |
| A09 | Infectious gastroenteritis and colitis, unspecified | 1,319,362 | 0.238 | 1,176,558 | 0.070 | 2,495,920 | 0.112 |
| B37 | Candidiasis of other sites | 555 | < 0.001 | 3,325 | < 0.001 | 3,880 | < 0.001 |
|  | **Total** | **1,400,044** | 0.253 | **1,267,404** | 0.076 | **2,667,448** | 0.120 |

ICD-10, International Classification of Diseases, 10th Revision.

**Table S6: Numbers of annual visits among normally healthy patients with acute infectious diarrhea alone by age group and sex**

| **Age group** | **Sex** | **2013** | **2014** | **2015** | **2016** | **2017** | **2018** | **Total** |
| --- | --- | --- | --- | --- | --- | --- | --- | --- |
| 0–17 years | Male | 29,534 | 25,478 | 26,541 | 27,985 | 24,083 | 22,402 | 156,023 |
|  | Female | 23,010 | 19,602 | 20,769 | 21,014 | 18,554 | 17,794 | 120,743 |
| 18–65 years | Male | 21,488 | 18,810 | 21,078 | 22,753 | 20,472 | 19,306 | 123,907 |
|  | Female | 16,654 | 12,707 | 13,914 | 14,515 | 12,723 | 11,298 | 81,811 |

**References**

1. Imafuku S, Matsuki T, Mizukami A, et al. Burden of herpes zoster in the Japanese population with immunocompromised/chronic disease conditions: results from a cohort study claims database from 2005–2014. *Dermatol Ther (Heidelb)* 2019;9:117-133.

2. Yanni EA, Ferreira G, Guennec M, et al. Burden of herpes zoster in 16 selected immunocompromised populations in England: a cohort study in the Clinical Practice Research Datalink 2000-2012. *BMJ Open* 2018;8:20528.
